# From Clusters to Bulk: Searching for the Stability Crossover between I_h_ and I_c_ via AVBMC Simulations

**DOI:** 10.1021/acs.jpcc.6c03068

**Published:** 2026-07-09

**Authors:** Bin Chen

**Affiliations:** Department of Chemistry, 5779 Louisiana State University, Baton Rouge, Louisiana 70803-1804, United States

## Abstract

Among the more than 20 known crystalline phases of ice, hexagonal (I_h_) and cubic (I_c_) ice are the dominant polymorphs at ambient pressure. Their relative thermodynamic stability and competition have been the subject of extensive investigation. This work presents a systematic study of the free energy landscape spanning from small clusters to the bulk for both polymorphs using a lattice-based aggregation-volume-bias Monte Carlo (LB-AVBMC) approach. Simulations were performed with the TIP4*P*/2005 water model at 300 K. Although this temperature exceeds the model’s melting point, the lattice constraint stabilizes crystalline clusters even at small sizes. It was found that the relative stability of I_h_ and I_c_ exhibits nontrivial size dependence, with their free energies interweaving in the small-cluster regime. Nucleation free energies computed for large clusters were analyzed within the Tolman-corrected classical nucleation theory framework to extrapolate bulk chemical potentials. While I_h_ is more stable than I_c_, both crystalline phases remain thermodynamically metastable relative to the liquid phase at 300 K. By combining bulk chemical potentials with enthalpy data obtained from additional *NpT* simulations, the melting temperature is estimated to be around 250 K, in good agreement with previous studies. The enthalpy difference between I_h_ and I_c_ is approximately 20 J/mol at 300 K and decreases with decreasing temperature.

## Graphical abstract

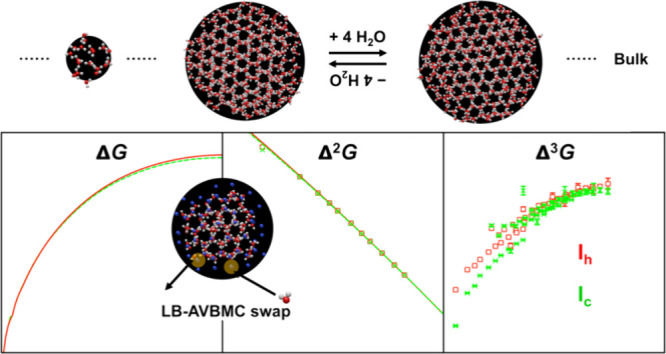

## 1 Introduction

Ice exhibits a remarkably rich phase behavior, with more than 20 known crystalline polymorphs.^1^ Among these, hexagonal (I_h_) and cubic (I_c_) ice are the two dominant forms at ambient pressure. I_h_ is well established as the thermodynamically stable phase under atmospheric conditions, whereas I_c_ is generally regarded as a metastable form that appears at low temperatures and transforms irreversibly to I_h_ upon heating.^2–4^ Since the identification of I_c_, the relative stability of these two polymorphs and their roles in ice nucleation have attracted sustained interest.^3–6^

Bulk-phase studies indicate that the energetic difference between I_h_ and I_c_ is extremely small. For example, calorimetric measurements of the I_c_-to-I_h_ transition report an enthalpy change of −37.7 ± 2.3 J/mol at 226 K.^7^ Despite the greater stability of I_h_ in the bulk, I_c_—or stacking-disordered mixtures of I_h_ and I_c_—is frequently observed during rapid cooling or in small droplets.^6,8–10^ This observation suggests the possibility of a size-dependent crossover in thermodynamic stability between the two polymorphs. A systematic characterization of the free energy landscape across cluster sizes is therefore essential to assess this possibility.

Experimental access to the structural and thermodynamic properties of small ice clusters remains limited.^11^ Computer simulations provide a valuable alternative for probing early stage crystallites, particularly during nucleation, although they are often constrained by computational cost.^12–14^ In this context, theoretical frameworks such as classical nucleation theory (CNT) can serve as useful complements.^15^ While small clusters deviate significantly from the assumptions of CNT, larger clusters are often well described by such models. This has served as the basis for using the cluster-based approaches to extrapolate the thermodynamic properties of bulk systems. For instance, simulations employing aggregation-volume-bias Monte Carlo (AVBMC)^16,17^ have shown that by interpreting the size dependence of nucleation free energies for sufficiently large liquid water clusters under the Tolman-corrected CNT framework, both the saturated gas-phase density and the bulk liquid–vapor surface tension obtained were found in excellent agreement with previous studies using conventional bulk-phase based approaches.^18–20^ It is therefore of interest to examine whether the same holds for crystalline clusters.

In this work, a lattice-based variant of AVBMC (LB-AVBMC)^21,22^ is applied to investigate the free energy landscapes of I_h_ and I_c_ from small clusters to the bulk using the TIP4P/2005 water model.^23^ The simulation methodology is described in Section 2, followed by results and discussion in Section 3 and conclusions in Section 4.

## 2 Methods

The lattice-based aggregation-volume-bias Monte Carlo (LB-AVBMC)^21,22^ method is designed specifically for crystalline cluster simulations. Its key distinction from conventional AVBMC lies in the definition of the local sampling volume used in particle swap moves. In LB-AVBMC, this volume (denoted *V*_in_) is defined as a sphere of radius *r* centered on a lattice site associated with a target molecule (see Figure 1). In contrast, conventional AVBMC^16,17^ defines *V*_in_ as a larger sphere of radius *R* centered on the molecule itself. The use of a smaller, lattice-centered volume constrains particles to remain near lattice positions characteristic of a given polymorph. Without this restriction, clusters generated by conventional AVBMC tend to be disordered even under conditions where ordered structures are thermodynamically favored.^24^

**Figure 1:**
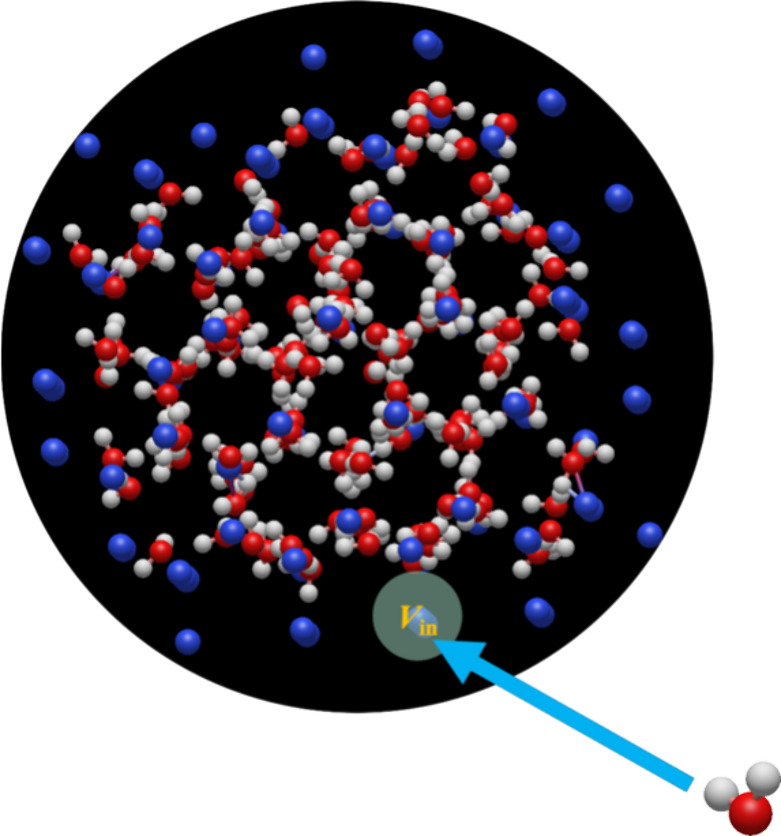
Schematic drawing of the volume (V_in_) used for particle insertion that is centered on one of the lattice sites neighboring to the lattice site that the target particle belongs to. Color notations: oxygen (red), hydrogen (white), and lattice site (blue).

As in conventional AVBMC, clusters in LB-AVBMC are defined using the Stillinger^25^ criterion, with additional constraints imposed by the lattice framework. Specifically, each particle must remain within a distance *r* of its assigned lattice site, and no two particles may occupy the same site. A cluster is defined as a group of molecules in which each molecule has at least one neighboring molecule within the group. Two molecules are considered neighbors if their corresponding lattice sites are adjacent.

In simulations of ice, each water molecule is assigned a unique lattice site based on the position of its oxygen atom, which must remain within *V*_in_. Trial moves that displace the oxygen atom outside this region are rejected. Each molecule has four neighboring lattice sites, consistent with tetrahedral coordination. The occupancy of these neighboring sites is tracked to facilitate particle insertion and deletion moves.

Particle swap moves employ energy-biased schemes^19,26–28^ in both the selection of the target molecule and, for deletion moves, the choice of the molecule to be removed. In deletion, the target molecule is first chosen based on the maximum energy among its neighbors, after which one of its neighbors—preferentially the one with the highest energy—is removed, thereby favoring the elimination of high-energy configurations. In insertion, the target molecule is selected based on its own energy, with higher-energy sites more likely to be chosen, which helps avoid selecting fully occupied local environments. Once a target site is selected, insertion proceeds by placing a randomly oriented water molecule within *V*_in_ of an empty neighboring lattice site.

To enhance insertion acceptance probabilities, a configurational-bias Monte Carlo (CBMC)^29–34^ scheme is employed. In contrast to a sequential growth procedure, insertion is performed in a single step. Specifically, *n*_trial_ trial configurations (here, *n*_trial_ = 10) are generated, each consisting of a randomly selected oxygen position within *V*_in_ and an independently assigned random orientation for the water molecule. Thus, each trial represents a complete molecular configuration. Selection among these trial configurations is carried out using the Rosenbluth^35^ weighting scheme, in which each trial is weighted by its Boltzmann factor. A configuration is then chosen with probability proportional to its weight, thereby biasing the sampling toward low-energy configurations while preserving detailed balance by including the Rosenbluth weight in the final Metropolis^36^ acceptance criterion.

Additional methodological details, including energy-biasing schemes, umbrella sampling (US)^37^ for uniform cluster-size sampling, and the combined AVBMC/CBMC implementation, are provided in prior publications^19^ and the Supporting Information.

Nucleation free energies Δ*G*(*n*) were analyzed using the second order finite-difference Δ^2^ *G*(*n*) = Δ*G*(*n* + 2) – Δ*G*(*n* – 2) for cluster sizes up to 2000 molecules. The size dependence of Δ^2^ *G*(*n*) was interpreted within the framework of Tolman-corrected classical nucleation theory,^19^ from which the chemical potential difference Δμ, the planar surface tension γ^∞^, and the Tolman length δ can be extrapolated, which, in turn, allows for reconstruction of the free energy landscape from finite clusters to the bulk limit (see Supporting Information for more details).

All simulations were performed using the grand canonical ensemble where the cluster is physically isolated but thermodynamically connected with an ideal gas phase. All intermolecular interactions are included in the calculation. In addition to particle swap moves, translational moves and rotational moves around the oxygen atom are employed in these calculations with equal probability. The maximum displacement was fixed at 0.3 Å for translation and 0.4 radius for rotation. Both exhibit acceptance rates of approximately 50%. The acceptance rate for particle swap increases from around 9% for clusters smaller than 100 to around 10% for clusters larger than 200, comparable to what has been previously found for liquid clusters.^19^ For each cluster size, short simulation runs under high supersaturation conditions were used to grow the clusters to the target size range, followed by another few short runs to converge the biasing potentials used in US (so that clusters are sampled uniformly—within a few percent), then by long production runs consisting of a total of O (10^11^) moves collected from 20 to 80 independent simulation runs. The production runs were grouped into five blocks of equal length, from which the standard uncertainties were estimated. For I_c_, at a few cluster sizes, simulations were also performed using an initially perfect cubic ice cluster, obtained from the bulk-phase simulations using periodic boundary conditions (PBC),^38^ to examine how the simulation results would be affected by the starting configuration.

## 3 Results and Discussion

### Dependence of the Simulation Results on the Initial Configuration

Before presenting the main results, it is important to examine how the starting configuration would affect the simulation results such as Δ^2^ *G* values. For I_c_, at a few sizes, the clusters were prepared in two different ways. They were either grown from those clusters at adjacent sizes (called as built) or prepared from the bulk-phase simulations under PBC (called as cube). In the former, the convergence of the free energy result (that is, when the frequency reaches uniformity for all clusters within a few percent during two consecutive runs and when the Δ^2^ *G* result starts to fluctuate around a certain value) can be achieved within a small fractional cost of the entire simulation that includes the production runs. In the latter, the convergence takes significantly longer. Also, as expected, the larger the cluster size, the longer it takes. For example, at 216, the convergence runs using the cube setup consumed more than 10% of the cost. At 512, the convergence run consumed more than 50% of the cost. It could be because the initial perfect cube where all particles are close to their lattice positions is far from the final sphere-like cluster structure with particles on the cluster surface more likely to wander away from their lattice positions. The Δ^2^ *G* value was found to increase monotonically during these runs. Once convergence was achieved, production runs were performed and the Δ^2^ *G* results yielded from these two different set-ups agree with each other. For example, at 216, Δ^2^ *G* was found to be 1.556 ± 0.003 *k*_B_ *T* when using the built setup vs 1.557 ± 0.003 *k*_B_ *T* using cube. All these simulations were performed with *r* = 1 Å. Once equilibrated large cluster structures have been obtained, they can be trimmed to a nearly spherical cluster of a smaller size (called as sphere). By contrast, when using the sphere setup, convergence can be more easily achieved for both I_h_ and I_c_. For most cluster sizes, initial configurations were prepared using at least two different ways, i.e., either built or sphere, to check the consistency of the simulation results obtained.

### Dependence of the Simulation Results on the Parameter *r*

For I_h_, in addition to *r* = 1 Å, simulations were also carried out using *r* = 1.2 and 1.38 Å. 1.38 Å corresponds approximately to the largest nonoverlapping local volume permitted by the lattice geometry since the nearest lattice sites have a separation of ∼2.77 Å. Figure 2 compares the Δ^2^ *G* values obtained. Like what has been shown previously for the Lennard-Jones (LJ) system,^21^ the Δ^2^ *G* data were found to be dependent on this parameter. The larger the *r* value, the lower the Δ^2^ *G* value. On the other hand, the larger the cluster size, the smaller the deviation on the Δ^2^ *G* values obtained using these different *r* values. It appears that they would all approach a similar value toward the infinite cluster size (or the bulk limit). Indeed, the Tolman-corrected CNT fit to Δ^2^ *G* over the cluster size range of 80 to 2000 leads to an intercept value (in units of *k*_B_ *T*) of −4.85 ± 0.25, −4.84 ± 0.05, and −4.87 ± 0.02, at *r* = 1.0, 1.2, and 1.38 Å, respectively. This intercept is related to the chemical potential difference Δμ between the hypothetical ideal gas phase reservoir used in the grand canonical simulation and the bulk phase. More exactly, it is equal to 4 times Δμ since Δ^2^ *G* is defined as Δ*G*(*n* + 2) – Δ*G*(*n* – 2), and can be interpreted as the reversible work in the process of adding 4 monomers to the cluster, while Δμ is exactly the reversible work of adding a single monomer to the bulk phase (which corresponds to an infinitely large cluster). Also shown in this figure are the Δ^2^ *G* values obtained previously for liquid clusters^19^ at the same simulation condition with its Tolman-corrected CNT fit. Throughout the entire cluster size range (not just those sizes investigated via the simulations since the Tolman-corrected CNT fit allows for the examination of the whole free energy landscape including the infinite size), Δ^2^ *G* values were consistently lower for liquid clusters than for I_h_ clusters. This result is expected since the melting point for the TIP4P/2005 model was found to range from 248 to 250 K,^39–43^ which is below 300 K used for these simulations.

**Figure 2:**
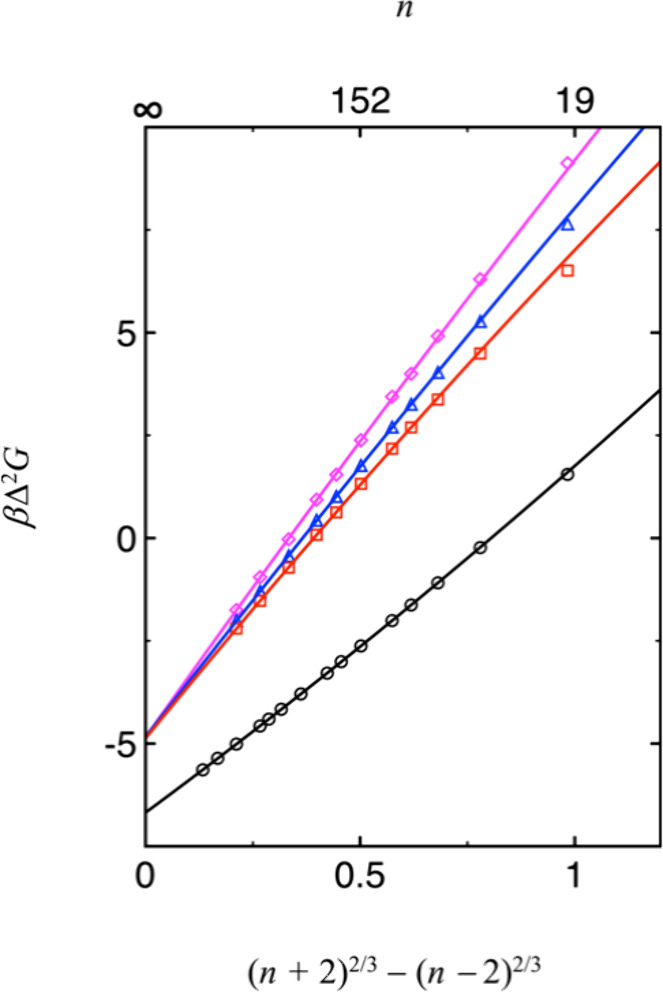
Δ^2^ *G* results for liquid water (circles) and I_h_ using *r* = 1.0 (diamonds), 1.2 (triangles), and 1.38 Å (squares) at *T* = 300 K and ρ_v_ = 1 × 10^–6^ molecule Å^–3^ with Tolman-corrected CNT fits (lines).

### Dependence of the Interpreted Δμ on the Range of Cluster Sizes Used in the Tolman-Corrected CNT Fit

Figure 3 shows how the extrapolated Δμ depends on the range of cluster sizes included in the Tolman-corrected CNT fit. Compared to the liquid system, the solid system displays a much larger scattering. In general, the larger the range of cluster sizes used in the fit, the more stable the Δμ value estimated irrespective of *r*. When *r* is large (at either 1.2 or 1.38 Å), inclusion of small clusters containing 20 molecules clearly leads to lower Δμ values, presumably because the use of a larger *r* may not be sufficient to keep these small clusters as solid-like as larger ones or similar-sized clusters sampled with a smaller *r*. Thus, when using a larger *r* value, these small clusters should be excluded in using this fit to estimate Δμ, or if one wants to use clusters as small as possible for this interpretation, a smaller *r* is recommended. On the other hand, the use of a smaller *r* can lead to larger uncertainty/oscillation on the interpreted Δμ, when a small range of larger clusters are included, probably because the less fluid-like surface can be more challenging to sample, especially for larger clusters. A close examination of the data obtained for liquid clusters shows a similar behavior, e.g., inclusion of clusters containing 20 molecules leads to lower Δμ, except that the oscillation/uncertainty is much smaller when using a comparable simulation length.

**Figure 3:**
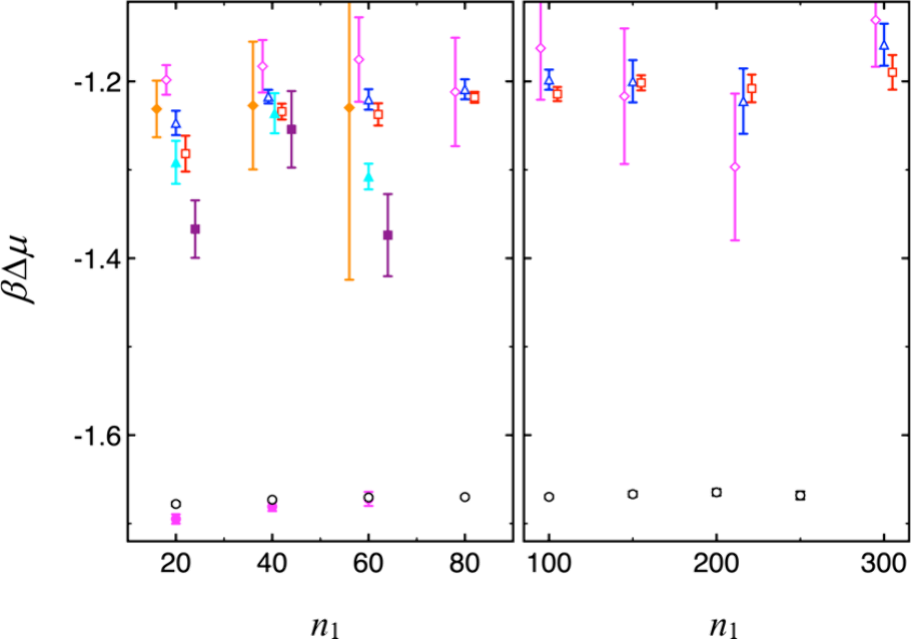
Δμ in units of *k*_B_ *T* obtained from weighted fits to the Tolman-corrected CNT expression of Δ^2^ *G* data over different cluster size ranges [*n*_1_, *n*_2_], plotted as a function of *n*_1_. Results are shown for *n*_2_ = 2000 (open symbols) and 216 (filled symbols) for liquid water (circles) and I_h_ using *r* = 1.0 (diamonds), 1.2 (triangles), and 1.38 Å (squares).

For the liquid system, using the Δμ value extrapolated from the fit to the size range between 80 and 2000, the saturated vapor phase density has been found to be in excellent agreement with previous work using bulk-phase based simulation approaches.^19,44,45^ For the solid system even with the largest *r* of 1.38 Å, the uncertainty on Δμ is significantly larger than that found for the liquid system if the same simulation length consisting of about 1 × 10^11^ Monte Carlo moves was used. To reduce the uncertainty, the simulations for I_h_ at *r* = 1.38 Å were extended to be about five times longer and a similar simulation length was used on I_c_ using *r* = 1.38 Å.

### Comparison of the Thermodynamic Stability between I_h_ and I_c_

Figure 4 compares the Δ^2^ *G* results obtained at *r* = 1.38 Å for I_c_ to I_h_. Except at *n* = 20, where I_c_ shows a noticeably lower Δ^2^ *G* than I_h_, the results obtained for these two systems are similar. A close examination of the Δ^2^ *G* values (see Table S1) does indicate that I_c_ are thermodynamically favored over I_h_ at small clusters, but this preference diminishes toward large cluster sizes. As a result, the Tolman-corrected CNT fits to the data generally leads to a similar Δμ value between I_c_ and I_h_ (see Figure 5), and in some cases (e.g., using the size range from 216 to 2000), a noticeably lower value for I_h_, suggesting that toward the bulk limit, I_h_ is thermodynamically more stable than I_c_, but both are metastable at this temperature condition because the liquid phase has the lowest chemical potential. It should be noted that the error bars in Figure 5 represent reduced chi-squared errors that include the goodness of fit. Even with such a long simulation, the data exhibit quite large uncertainties/oscillations. The strong resemblance of the oscillatory behavior between the two sets of data obtained from both the long and the short simulation runs for I_h_ indicates that the errors are systematic, most likely because certain cluster sizes may exhibit enhanced structural stability relative to neighboring sizes, analogous to magic-size effects observed in finite systems.

**Figure 4:**
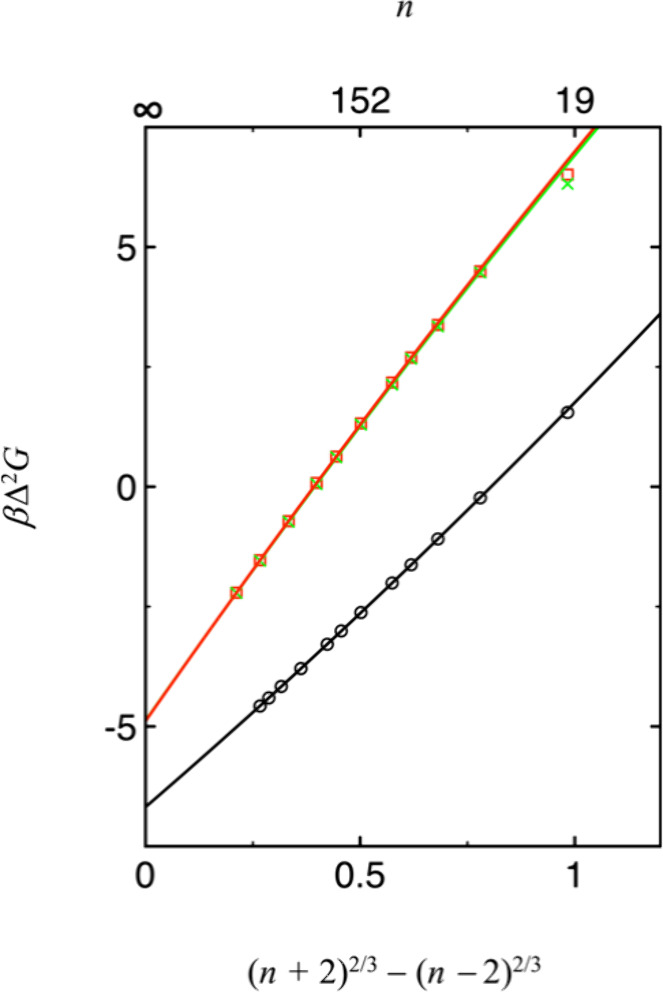
Δ^2^ *G* results for I_h_ (squares) and I_c_ (crosses) using *r* = 1.38 Å at *T* = 300 K and ρ_v_ = 1 × 10^–6^ molecule Å^–3^ with Tolman-corrected CNT fits (lines). For comparison, the results obtained for liquid water are also shown.

**Figure 5:**
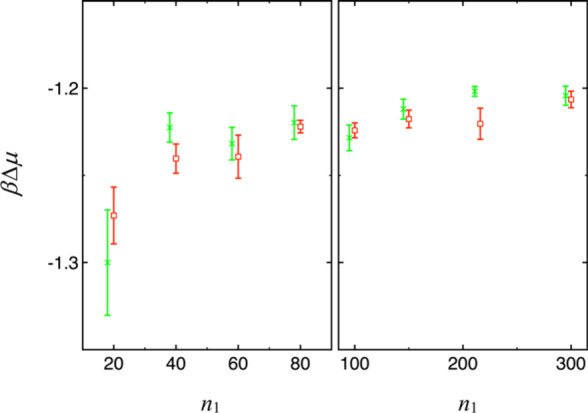
Δμ in units of *k*_B_ *T* obtained from weighted fits to the Tolman-corrected CNT expression of the Δ^2^ *G* data over different cluster size ranges [*n*_1_, 2000], plotted as a function of *n*_1_ for I_h_ (squares) and I_c_ (crosses).

Structural analysis with Chill+^46^ shows a small fraction of eclipsed bonds present in I_c_ clusters. Chill+ has been frequently used to characterize the local structure of a given water molecule or the water–water bond formed between its first-solvation shell neighbors and this reference molecule via the Steinhardt order parameter,^47,48^ particularly, *q*_3_. Based on the dot product value of *q*_3_ between a pair of neighboring water molecules, the water–water bond can be classified as a staggered or eclipsed bond. For bulk I_h_, each water molecule is surrounded by three staggered and one eclipsed water–water bond. For bulk I_c_, all four water–water bonds are staggered. The presence of a small amount of eclipsed bonds in I_c_ clusters is an indication of the tendency to form stacking dis-ordered ice and suggests that the use of distance constraint with a relatively large *r* value alone may be insufficient to constrain the structure to a particular polymorph. Although the use of the Chill+ algorithm can be problematic to characterize the structure of interfacial water molecules which are present for these cluster systems, the use of a small *r* does show that the amount of eclipsed bonds can be reduced significantly (see Figure S1), forming a purer I_c_ structure. Even with the smallest *r*, I_c_ clusters were found slightly more stable than I_h_ ones, but their thermodynamic stability exhibits nontrivial size dependence, with their free energies interweaving in the small-cluster regime (see Figure 6). With increasing *r*, the thermodynamic stability of I_c_ over I_h_ is enhanced in the small-cluster regime, most likely due to an increased mixing of I_c_ with other structures.

**Figure 6:**
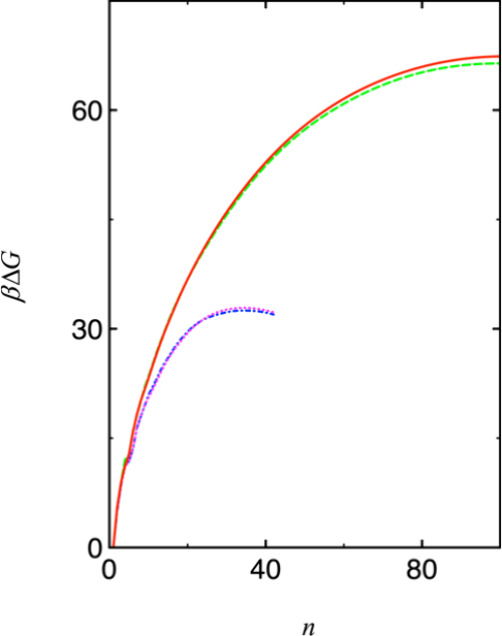
Δ*G* results obtained using *r* = 1.38 Å at ρ_v_ = 1.7 × 10^–6^ molecule Å^–3^ for I_h_ (red solid) and I_c_ (green dashed) or using *r* = 1.0 Å at ρ_v_ = 5.42 × 10^–6^ molecule Å^–3^ for I_h_ (magenta dotted) and I_c_ (blue dashed-dotted). Errors are smaller than the line width.

### Surface Tension and Tolman Length

In addition to Δμ, the Tolman-corrected CNT fits to Δ^2^ *G* yield the surface tension of the cluster-vapor interface for an infinitely large cluster under the same constraint, γ^∞^, and the Tolman length, δ.^49^ When a sufficient large *r* was used, the interpreted surface tension for a LJ solid system^50^ and for various liquid systems including water^19,20^ has been found to agree with the planar surface tension value calculated using other bulk-phase based approaches. Figure 7 shows how the extrapolated γ^∞^ depends on both the range of cluster sizes included in the Tolman-corrected CNT fit and *r* for I_h_. Like the chemical potential results shown in Figure 3, using the smallest *r* of 1.0 Å causes large oscillation and uncertainty of the interpreted γ^∞^. Even with this large oscillation/uncertainty, there is a systematic overestimation of the surface tension because of the less fluid-like surface. The results obtained at *r* = 1.2 Å are relatively more stable with much smaller errors. They are also much closer to those at 1.38 Å. There is lack of literature data for ice-vapor interfacial tension. For TIP4P/2005, the ice/water interfacial tension was estimated to be about 29.8 mN/m.^51^ This combined with the liquid water/vapor surface tension of this model leads to an estimate of the ice/vapor surface tension close to 100 mN/m. Also included in this figure are the results obtained for I_c_ with *r* = 1.38 Å. Compared to I_h_, a lower surface tension was generally estimated for I_c_, which is the thermodynamic driving force to favor I_c_ over I_h_ at small cluster sizes, like BCC vs FCC found for LJ.^50,52^

**Figure 7:**
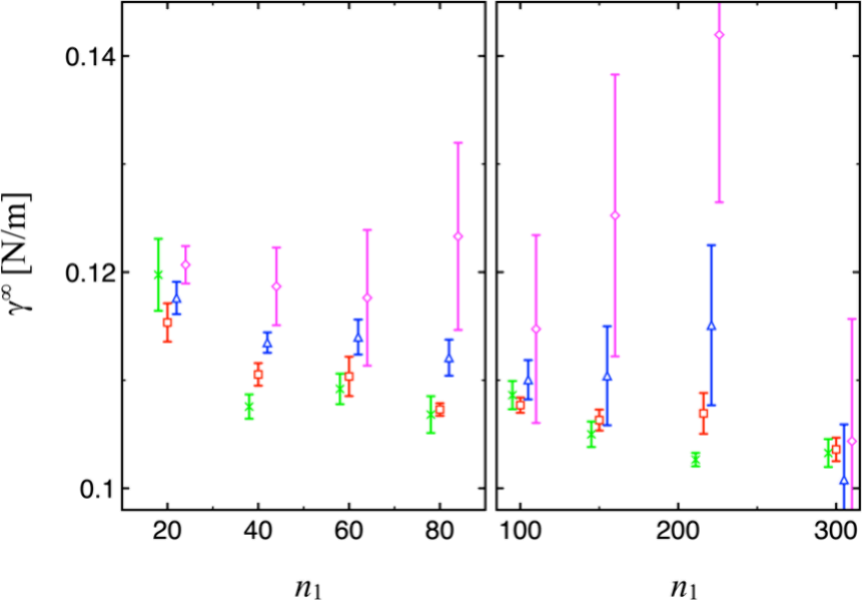
γ^∞^ in units of N/m obtained from weighted fits to the Tolman-corrected CNT expression of the Δ^2^ *G* data over different cluster size ranges [*n*_1_, 2000], plotted as a function of *n*_1_. Results are shown for I_h_ using *r* = 1.0 (diamonds), 1.2 (triangles), and 1.38 Å (squares). The results obtained for I_c_ using *r* = 1.38 Å are shown as crosses.

Figure 8 shows how the extrapolated δ depends on the range of cluster sizes included in the Tolman-corrected CNT fit for both I_h_ and I_c_. In contrast to a negative value that has been found previously for liquid clusters (including both LJ and water),^18–20^ δ can take a positive value for both I_h_ and I_c_, and more positive when a larger *r* is used and/or small clusters are included in this analysis. A positive effective Tolman length from these fitted results are consistent with the Δ^3^ *G* plots (see Figure 9), which display a slope opposite to what was found previously for liquid water clusters. While for liquid water clusters surface tension decreases with the increasing cluster size, the present analysis for both ice polymorphs suggests an increase in effective surface tension with increasing cluster size, as the surface become less fluid-like with a more solid-like structure forming in the interior of the cluster, which is reminiscent to the transition from fractal to more compact structure by LJ liquid clusters during the initial growth. Consequently, the Δ^3^ *G* results of these systems in the small cluster size range resemble each other. However, for the liquid LJ cluster system, the Δ^3^ *G* results in the larger cluster size range show an opposite slope. In fact, a negative δ value of −0.0066 ± 0.0013 was interpreted based on Δ^3^ *G* results obtained for larger clusters containing up to 8000 particles.^18^

**Figure 8:**
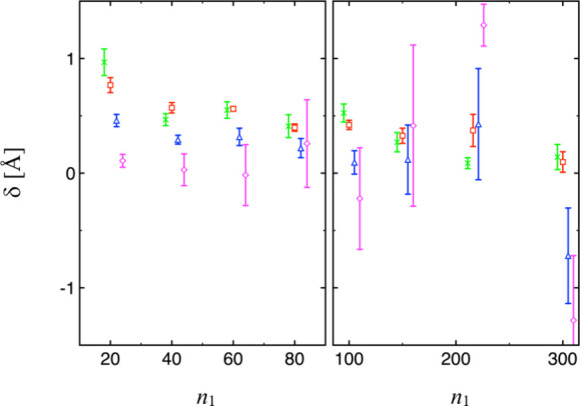
δ in units of Å obtained from weighted fits to the Tolman-corrected CNT expression of the Δ^2^ *G* data over different cluster size ranges [*n*_1_, 2000], plotted as a function of *n*_1_. Results are shown for I_h_ using *r* = 1.0 (diamonds), 1.2 (triangles), and 1.38 Å (squares). The results obtained for I_c_ using *r* = 1.38 Å are shown as crosses.

**Figure 9:**
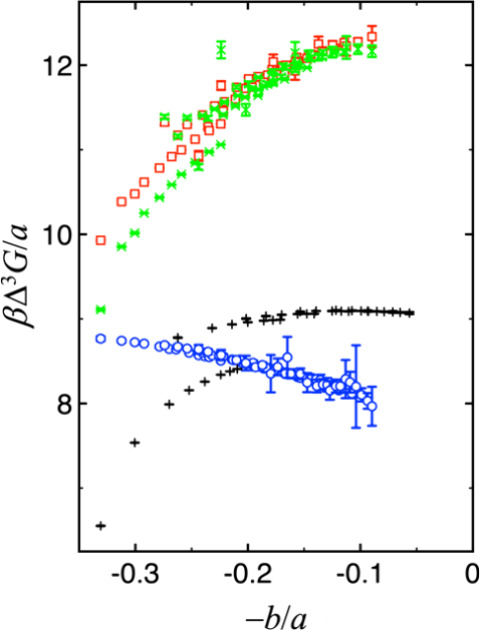
Δ^3^ *G*(*n*, *m*)/*a*(*n*, *m*) in units of *k*_B_ *T* as a function of *b*(*n*, *m*)/*a*(*n*, *m*) obtained for I_h_ (red), I_c_ (green), liquid water (blue), and LJ (black) with Δ^3^ *G*(*n*, *m*) = Δ^2^ *G*(*n*) – Δ^2^ *G*(*m*), *a*(*n*, *m*) = (*n* + 2)^2/3^ – (*n* – 2)^2/3^ – [(*m* + 2)^2/3^ – (*m* – 2)^2/3^], and *b*(*n*, *m*) = (*n* + 2)^1/3^ – (*n* – 2)^1/3^ – [(*m* + 2)^1/3^ – (*m* – 2)^1/3^]. Under the Tolman-corrected CNT framework,^19^ the slope of this plot is governed by δ.

The sign of δ and how surface tension would change with the cluster size have been highly debated,^53–57^ especially on liquid systems for which the surface tension can be more easily calculated. For solids, surface tension is more challenging to obtain than liquids,^58^ but the size dependence of interfacial free energy has long been recognized as a limitation of CNT.^59,60^ The combined simulation/Tolman-corrected CNT framework presented here provides a route to extrapolate both γ and δ for solids, which can help to resolve controversial issues on these important properties.

### Influence of Quasi-Liquid Layers on Surface Tension and Tolman Length

One limitation of the present LB-AVBMC framework is that molecules are constrained to remain within a distance *r* of their assigned lattice sites. Consequently, large-amplitude molecular motions are suppressed, and a realistic quasi-liquid layer (QLL) cannot form at the cluster surface. The extracted surface tensions and Tolman lengths should therefore be interpreted as effective quantities associated with the constrained crystalline clusters employed in the LB-AVBMC framework, especially when using small clusters or small *r*. Regarding the influence of the QLL on surface tension (free energy), it has been shown that, for the LJ system, this approach—when using sufficiently large *r*—can reproduce results consistent with bulk-phase studies of surface tension.^50^ For water, however, reliable surface tension data in this regime are lacking. At 300 K, the conventional bulk-phase approach is not applicable, since ice would completely melt under these conditions.

Although a direct comparison is therefore unlikely, the temperature dependence of surface tension can still be used to extrapolate its value at 300 K. In principle, both the conventional slab-based approach and the present LB-AVBMC approach could be adapted by constraining part of the system to a lattice while allowing a variable amount of surrounding liquid. Such calculations could provide a more direct assessment of how the QLL modifies the interfacial free energy. However, the primary focus of the present work is on thermodynamic stability rather than the microscopic origin of the surface free energy. A systematic investigation of how QLL formation influences the interpretation of surface tension and Tolman lengths would therefore be an interesting direction for future work.

### Determination of the Melting Point

Melting point is defined as the condition when the chemical potential of the solid phase is equal to that of the liquid phase. To determine this property, or the temperature when the chemical potentials of these two phases are equal, the Gibbs–Helmholtz^61^ equation is used. Additional *NpT* simulations for both solid and liquid water were performed to obtain the enthalpic data needed by this equation.

It has been found previously for TIP4P/2005 I_h_ and I_c_ have nearly the same internal energy within the temperature range from 200 to 250 K.^5^ Indeed, the *NpT* simulations yielded almost identical values for these two phases at low temperatures (see Figure 10). However, at high temperatures, the internal energy of I_h_ is slightly lower than that of I_c_. For instance, at 300 K the internal energy was found to be −53.972 ± 0.008 kJ/mol for I_h_ vs −53.948 ± 0.011 kJ/mol for I_c_, a difference of 24 J/mol. Similarly, these two phases have nearly identical densities at low temperatures but show small differences at high temperatures with I_c_ becoming slightly more densely packed than I_h_ (see Table S2). These *NpT* simulations were carried out using 1024 particles with an orthorhombic box for I_h_ and 1000 particles with a cubic box for I_c_.

**Figure 10:**
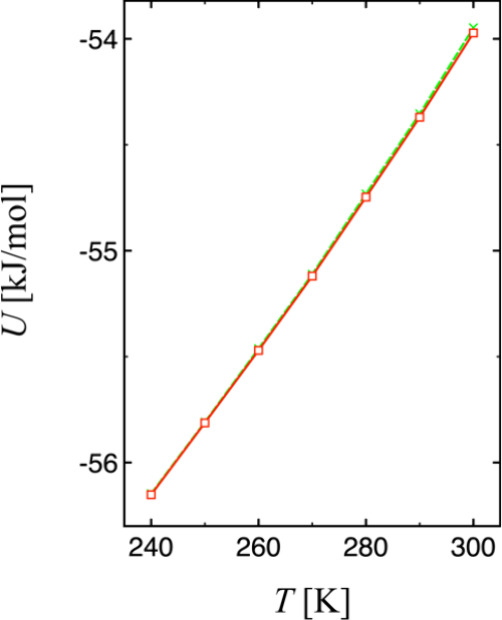
Internal energy in units of kJ/mol as a function of temperature for I_h_ (red) and I_c_ (green).

For both, the intermolecular interactions were truncated at 14 Å. While tail corrections are included for the Lennard-Jones part of the interactions, Ewald sum with a kappa value of 0.23 Å^–1^ and a tinfoil boundary condition are used to deal with the long-range electrostatic interactions.^38^ For both, 20 different proton-disordered structures were generated from the Genice tool.^62^ To examine whether the simulation results would be affected by the initial configurations, another set of 20 different structures were produced from this tool. This new set of simulations yielded an average internal energy of −53.986 ± 0.006 kJ/mol for I_h_ vs −53.960 ± 0.017 kJ/mol for I_c_ at 300 K. Consistent with the previous set of simulations, I_h_ was found lower in energy than I_c_ by a comparable amount of 26 J/mol. This amount of difference compares well with recent calorimetric measurements of the I_c_-to-I_h_ transition, which reported an enthalpy change of −37.7 ± 2.3 J/mol at 226 K.^7^

The enthalpy change from liquid water to I_h_ can be calculated from the energy and density information obtained for these two phases. When integrating the Gibbs–Helmholtz equation (to determine the melting point, or at which temperature the chemical potential difference between these two phases would vanish), the enthalpy change was interpreted to be linearly dependent on the temperature using the enthalpy data obtained at adjacent temperatures. For I_h_, the melting point was found to be 249.7 ± 0.5 K when using the size range from 80 to 2000, compared to previously reported literature values, e.g., 248.25 ± 0.75 K in ref 41. and 250.25 ± 0.25 K in ref 43. This validates the chemical potential extrapolated and the general applicability of this combined simulation/Tolman-corrected CNT framework to both solid and liquid systems. In addition, the melting point for I_c_ is found to be generally lower than I_h_ by up to 2 K (see Table S3). No other melting point calculations have been attempted previously for this model on I_c_. Via the Einstein crystal method,^63,64^ Zaragoza et al.^5^ found a lower chemical potential for ice I_h_ than I_c_ at 200 K, but the difference is within the error of the calculations. Using a deep neural network potential trained on ab initio calculations, Piaggi et al.^3^ found a lower melting point for I_c_ than I_h_ by a few Kelvins.

## 4 Conclusions

In this work, the lattice-based aggregation-volume-bias Monte Carlo method was used to investigate the nucleation free energy landscapes of I_h_ and I_c_ using the TIP4P/2005 water model. The lattice constraint stabilizes crystalline clusters over a broad size range, enabling systematic examination of the evolution of cluster thermodynamics from small clusters toward the bulk limit.

The results reveal pronounced size-dependent behavior in the small-cluster regime, where the relative stability of I_h_ and I_c_ alternates with cluster size. At larger sizes, the free energy landscapes are well described by Tolman-corrected CNT, allowing extrapolation toward the bulk limit. The extrapolated chemical potentials indicate that I_h_ is thermodynamically more stable than I_c_, while I_c_ exhibits lower effective surface free energies that enhance its stability at finite sizes.

Both crystalline phases remain thermodynamically metastable relative to the liquid phase at 300 K. Combining the extrapolated chemical potentials with enthalpy data from independent *NpT* simulations yields an estimated melting temperature of approximately 250 K for the TIP4P/2005 model, in good agreement with previous studies.

Overall, the present work demonstrates that LB-AVBMC combined with Tolman-corrected CNT provides an effective framework for connecting finite crystalline clusters with bulk thermodynamic behavior in polymorphic molecular systems.

The LB-AVBMC framework may also be adaptable to simulations of crystalline cluster growth in supercooled liquid water, providing a potential route for investigating polymorphism and interfacial thermodynamics during ice nucleation in more complex environments.

Finally, recent molecular simulation studies have provided atomistic insight into heterogeneous ice nucleation and the role of interfacial structure in controlling nucleation pathways and free-energy barriers.^65^ While the present work focuses on curvature-dependent interfacial free energies in crystalline clusters, both approaches highlight the central role of interfacial properties in governing ice nucleation behavior.

## Supplementary Material



## Notes

Additional methodological details of the LB-AVBMC simulations; derivation of the Tolman-corrected CNT expressions; nucleation free energies (Δ^2^ *G*); internal energies (*U*); densities (ρ); extrapolated chemical potentials (Δμ); melting point data (*T*_m_); and fraction of eclipsed bonds (PDF)
