# Supplementary material for: From Clusters to Bulk: Searching for the Stability Crossover between Ih and Ic via AVBMC Simulations
Source: J Phys Chem C Nanomater Interfaces. 2026 Jul 9;130(29):10436–44. doi: 10.1021/acs.jpcc.6c03068 (PMC13403301; doi:10.1021/acs.jpcc.6c03068)
Supplement: Supplementary file 1 [file jp6c03068_si_001.pdf]

Supporting Information for:

From Clusters to Bulk: Searching for the Stability Crossover between I_h and I_c via AVBMC Simulations

Bin Chen*

Department of Chemistry, Louisiana State University, Baton Rouge, Louisiana 70803-1804, USA

*Corresponding author: email: binchen@lsu.edu

Technical Details of the LB-AVBMC Method and Tolman-Corrected Classical Nucleation Theory

1. Simulation Methodology

1.1 Lattice-Based AVBMC Framework

Nucleation free energies (NFEs) were computed using a lattice-based aggregation-volume-bias Monte Carlo (LB-AVBMC) method,[1] which extends the conventional AVBMC framework to crystalline systems. In this approach, each molecule is associated with a predefined lattice site corresponding to a given polymorph, and particle moves are constrained within a local sampling volume V_{in} centered on that site.

Clusters are defined using a modified Stillinger[2] criterion, with the additional requirement that each molecule must remain within a distance r of its assigned lattice site and that no lattice site may be occupied by more than one molecule. Two molecules are considered connected if their corresponding lattice sites are adjacent.

1.2 AVBMC Particle Swap Moves

Particle insertion and deletion moves are performed using biased selection schemes[3] to enhance sampling efficiency.

For **deletion**, a target molecule is first selected based on the maximum interaction energy among its neighboring molecules, favoring interfacial regions. Subsequently, one of its neighbors—preferentially the one with the highest interaction energy—is selected for removal. This procedure biases the sampling toward eliminating high-energy configurations.

For **insertion**, a target molecule is selected based on its own interaction energy, with higher-energy molecules more likely to be chosen. This bias reduces the probability of selecting fully occupied local environments and increases the likelihood of successful insertion.

These energy-biased schemes follow the general AVBMC framework, adapted here for lattice-based crystalline clusters.

1.3 Configurational-Bias (CBMC) Insertion Scheme

To improve insertion efficiency, a configurational-bias Monte Carlo (CBMC)[4] scheme is employed. In this implementation, insertion is performed in a single step rather than through a sequential growth procedure. Specifically, each insertion attempt generates n_{trial} trial configurations, each consisting of a randomly selected oxygen position within V_{in} of the chosen lattice site and an independently assigned random orientation for the water molecule. Each trial therefore represents a complete molecular configuration.

Selection among these trial configurations is carried out using the Rosenbluth[5] weighting scheme, in which each configuration is weighted by its Boltzmann factor. The Rosenbluth weight is given by

$$W = \sum_{i=1}^{n_{\text{trial}}} \exp(-\beta E_i)$$

where E_i denotes the interaction energy of trial configuration i with its nearest neighbors. A configuration is selected with probability proportional to its Boltzmann weight, thereby favoring low-energy configurations.

The interaction energy beyond nearest neighbors ($\varepsilon^{\text{tail}}$) is subsequently incorporated in the final acceptance probability. This procedure preserves detailed balance while significantly reducing the cost of energy calculation for trial configurations.

1.4 Acceptance Criteria

Insertion and deletion moves are accepted according to grand canonical ensemble criteria. The acceptance probabilities include contributions from:

- the insertion volume V_{in},
- the Rosenbluth weight W,
- other factors that contribute to the probability of generating this move and the reversed move, including the choice of the target molecule in both directions ($P_{\text{target, ins}}$ and $P_{\text{target, rem}}$), the selection of the molecule for removal ($P_{\text{select, rem}}$), and the probability to pick an empty lattice site among N_{vac} vacant sites,
- the interaction energy beyond the nearest neighbors ($\varepsilon^{\text{tail}}$),
- and the effective chemical potential (or vapor density representation, ρ_{v}).

Details of these acceptance rules follow standard AVBMC formulations and are provided here for completeness:

- **Insertion:**

$$P_{\text{accept, ins}} = \min\left\{1,\ \frac{P_{\text{target, rem}} \cdot P_{\text{select, rem}} \cdot V_{\text{in}} \cdot N_{\text{vac}}}{P_{\text{target, ins}}} \cdot \frac{W}{n_{\text{trial}}} \cdot \exp(-\beta\varepsilon_{\text{tail}}) \cdot \rho_{\text{v}}\right\}$$

- **Deletion:**

$$P_{\text{accept, rem}} = \min\left\{1,\ \frac{P_{\text{target, ins}}}{P_{\text{target, rem}} \cdot P_{\text{select, rem}} \cdot V_{\text{in}} \cdot (N_{\text{vac}}+1)} \cdot \frac{n_{\text{trial}}}{W} \cdot \exp(\beta\varepsilon_{\text{tail}}) \cdot {\rho_{\text{v}}}^{-1}\right\}$$

1.5 Sampling Strategy

Umbrella sampling[6] was employed to ensure uniform sampling across cluster sizes. Biasing potentials (which will be also included in the acceptance rule) were iteratively refined to produce approximately flat cluster-size distributions.

Cluster sizes up to n = 2000 were sampled. Rather than computing NFEs for every cluster size, selected sizes were used to construct finite-difference free energy estimates, improving computational efficiency.

2. Tolman-Corrected Classical Nucleation Theory

2.1 Finite-Difference Representation of Nucleation Free Energy

The nucleation free energy $\Delta G(n)$ was analyzed using the second-order finite-difference:

$$\Delta^2 G(n) = \Delta G(n+2) - \Delta G(n-2)$$

2.2 Tolman-Corrected CNT Framework

Within this framework,[7] the free energy of a cluster of size n with a radius of R is expressed as

$$\Delta G(n) = -n\Delta\mu + \gamma(R)A(R)$$

where:

- $\Delta\mu$ is the chemical potential difference between phases,
- $\gamma(R)$ is the curvature-dependent surface tension,
- $A(R)$ is the surface area of the cluster.

The Tolman correction introduces a size dependence to the surface tension:[8]

$$\gamma(R) = \gamma^{\infty}\left(1 - \frac{2\delta}{R}\right)$$

where γ^{∞} is the planar surface tension and δ is the Tolman length.

2.3 Size-Based Representation

Using the bulk relation between cluster size and radius,

$$n = \frac{4\pi}{3}\rho R^3$$

the free energy expression can be rewritten as a function of n:

$$\Delta G(n) = -n\Delta\mu + An^{2/3} - Bn^{1/3}$$

where A and B are constants depending on γ^{∞}, δ, and the bulk density ρ.

2.4 Parameter Extraction

The finite-difference free energy data $\Delta^2 G(n)$ obtained from LB-AVBMC simulations were fitted to the Tolman-corrected CNT expression. This fitting procedure yields:

- the chemical potential difference $\Delta\mu$,
- the planar surface tension γ^{∞},
- and the Tolman length δ.

These parameters enable reconstruction of the full free energy landscape, including extrapolation to the bulk limit.

Tables containing nucleation free-energy data ($\Delta^2 G$), internal energies (U), densities (ρ), extrapolated chemical potential data ($\Delta\mu$), melting point data (T_m), and a figure on the fraction of eclipsed bonds

Table S1: $\Delta^2 G = \Delta G(n+2) - \Delta G(n-2)$ in units of $k_{\mathrm{B}}T$ as a function of cluster size n obtained at $T = 300$ K and $\rho_{\mathrm{v}} = 1 \times 10^{-6}$ molecule/$\mathrm{\AA}^3$ using $r = 1.38$ Å

n	$\mathrm{I_c}$	$\mathrm{I_h}$
20	6.3171 ± 0.0019	6.5107 ± 0.0030
40	4.4655 ± 0.0016	4.4925 ± 0.0018
60	3.3419 ± 0.0016	3.3750 ± 0.0026
80	2.6691 ± 0.0031	2.6941 ± 0.0017
100	2.1286 ± 0.0021	2.1723 ± 0.0014
150	1.2953 ± 0.0032	1.3205 ± 0.0028
216	0.6142 ± 0.0024	0.6288 ± 0.0030
300	0.0542 ± 0.0039	0.0788 ± 0.0031
512	-0.7323 ± 0.0042	-0.7177 ± 0.0040
1000	-1.5463 ± 0.0050	-1.5327 ± 0.0062
2000	-2.2152 ± 0.0033	-2.2115 ± 0.0040

Table S2: Internal energy U in kJ/mol and density ρ in g/ml from NpT simulations as function of temperature T in Kelvin. Numbers in parentheses correspond to the uncertainty.

	I_c		I_h		liquid	
T	U	ρ	U	ρ	U	ρ
240	−56.147(4)	0.92184(3)	−56.152(4)	0.92177(3)	−51.845(38)	0.9855(22)
250	−55.809(3)	0.92023(3)	−55.813(3)	0.92017(4)	−51.054(32)	0.9946(6)
260	−55.462(3)	0.91862(2)	−55.471(2)	0.91849(3)	−50.347(13)	0.9978(12)
270	−55.111(4)	0.91694(3)	−55.120(2)	0.91680(2)	−49.634(16)	1.0001(8)
280	−54.732(7)	0.91535(4)	−54.747(7)	0.91515(6)	−48.974(13)	0.9999(7)
290	−54.354(10)	0.91368(8)	−54.370(7)	0.91342(5)	−48.309(14)	0.9990(2)
300	−53.948(11)	0.91200(4)	−53.972(8)	0.91172(4)	−47.657(7)	0.9961(4)

Table S3: $\Delta\mu$ in units of $k_B T$ from weighted Tolman-corrected CNT fits to $\Delta^2 G$ over cluster size range $[n_1, 2000]$ obtained at $T = 300$ K and $\rho_v = 1 \times 10^{-6}$ molecule/$\mathring{A}^3$ using $r = 1.38\mathring{A}$, and the corresponding T_m interpreted from the Gibbs-Helmholtz equation.

	I_c		I_h	
n_1	$\Delta\mu$	T_m	$\Delta\mu$	T_m
20	−1.300 ± 0.030	258.1 ± 3.3	−1.273 ± 0.0016	255.2 ± 1.8
40	−1.223 ± 0.008	249.7 ± 0.9	−1.240 ± 0.008	251.6 ± 0.9
60	−1.232 ± 0.009	250.6 ± 1.0	−1.239 ± 0.012	251.5 ± 1.3
80	−1.220 ± 0.010	249.3 ± 1.1	−1.222 ± 0.004	249.7 ± 0.5
100	−1.228 ± 0.007	250.2 ± 0.8	−1.224 ± 0.004	249.9 ± 0.5
150	−1.212 ± 0.006	248.5 ± 0.7	−1.218 ± 0.005	249.2 ± 0.6
216	−1.202 ± 0.003	247.4 ± 0.4	−1.220 ± 0.009	249.4 ± 1.0
300	−1.204 ± 0.005	247.6 ± 0.6	−1.206 ± 0.004	247.9 ± 0.5

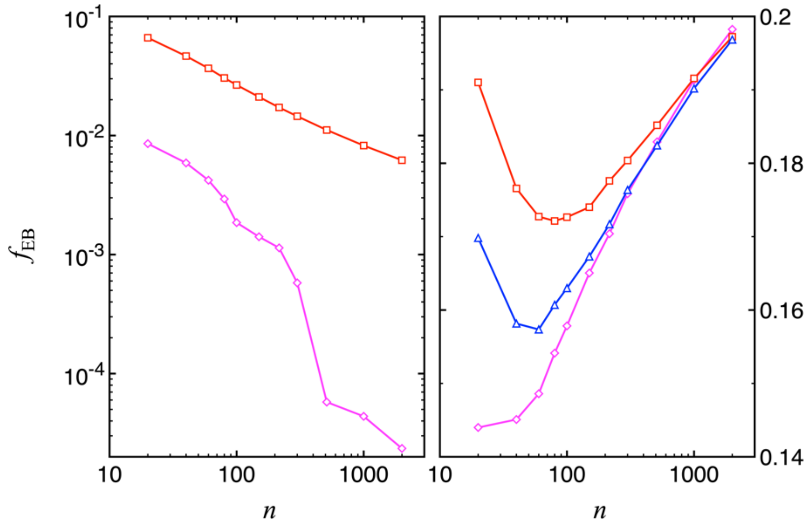

Figure S1. Fraction of eclipsed bonds as function of cluster size obtained for I_c (left) and I_h (right) using r = 1.0 Å (diamonds), 1.2 Å (triangles), and 1.38 Å (squares).

References

1. Chen, B. Extension of the Aggregation-Volume-Bias Monte Carlo Method to the Calculation of Phase Properties of Solid Systems: A Lattice-Based Cluster Approach. *J. Phys. Chem. A* **2022**, *126*, 5517–5524. https://doi.org/10.1021/acs.jpca.2c04333
2. Stillinger, F. H. Rigorous basis of the frenkel-band theory of association equilibrium. *J. Chem. Phys.* **1963**, *38*, 1486. https://doi.org/10.1063/1.1776907
3. Loeffler, T. D., Sepehri, A. & Chen, B. Improved Monte Carlo scheme for efficient particle transfer in heterogeneous systems in the grand canonical ensemble: Application to vapor–liquid nucleation. *J. Chem. Theory Comput.* **11**, 4023–4032 (2015). https://doi.org/10.1021/acs.jctc.5b00466
4. Siepmann, J. I.; Frenkel, D. Configurational bias Monte Carlo: A new sampling scheme for flexible chains. Mol. Phys. **1992**, *75*, 59-70. DOI: 10.1080/00268979200100061
5. Rosenbluth, M. N. & Rosenbluth, A. W. Monte Carlo calculation of the average extension of molecular chains. *J. Chem. Phys.* **23**, 356–359 (1955). https://doi.org/10.1063/1.1741967
6. Torrie, G. M. & Valleau, J. P. Nonphysical sampling distributions in Monte Carlo free-energy estimation: Umbrella sampling. *J. Comput. Phys.* **23**, 187–199 (1977). http://dx.doi.org/10.1016/0021-9991(77)90121-8
7. Chen, B.; Nguyen, N. M. N. Surface tension of infinitely planar surfaces from nucleation free energies: A comparison of Monte Carlo calculations and classical theories. *J. Chem. Theory Comput.* **2025**, *21*, 8051–8059. https://doi.org/10.1021/acs.jctc.5c01122
8. Tolman, R. C. The effect of droplet size on surface tension. *J. Chem. Phys.* **17**, 333–337 (1949). https://doi.org/10.1063/1.1747247
